# The influence of bruxism on post orthodontic direct anterior restorations integrity: a retrospective evaluation

**DOI:** 10.3389/froh.2026.1842684

**Published:** 2026-06-23

**Authors:** Ambra Sedran, Martina Ferrillo, Andrea Baldi, Andrea Roggia, Sara Peano, Allegra Comba, Aristea Cedrone, Nicola Scotti

**Affiliations:** 1 Department of Surgical Sciences, Dental School, University of Turin, Turin, Italy; 2 Dentistry Unit, Department of Health Sciences, University of Catanzaro “Magna Graecia”, Catanzaro, Italy; 3 Private Practitioner, Turin, Italy

**Keywords:** bruxism, direct restorations, fracture, muscle activity, SEM analysis, sEMG

## Abstract

**Objectives:**

The aim of this study was to evaluate the influence of bruxism on post orthodontic direct anterior restorations integrity.

**Material and methods:**

In this retrospective study, adult subjects who received additive composite restorations in maxillary anterior teeth after an orthodontic treatment were recruited. Study group consisted of patients who reported fracture of the additive composite restorations, whereas controls were recruited among the patients who reported success, according to the modified United States Public Health Service (USPHS) criteria. A bruxism evaluation was performed according to the Standardized Tool for the Assessment of Bruxism (STAB). A Scanning Electron Microscopy evaluation was conducted to obtain a fractographic analysis.

**Results:**

A total of 40 restorations from 20 patients (8 males and 12 females, mean aged 25.67 ± 4.77 years) were evaluated. The following electromyographic variables resulted to be significantly different between groups: PC (*p*-value = 0.003), TC (*p*-value = 0.003), MC (*p*-value = 0.002), and TMC (*p*-value = 0.000). The fractographic analysis revealed that the fractures originated at the site of traumatic contact with the antagonist tooth.

**Conclusions:**

At follow-up, patients with fractured anterior restorations showed significantly higher masseter muscle activity, evaluated in terms of phasic contractions, tonic contractions, mixed contractions, and total masseter contractions using a portable sEMG portable device. The fractographic analysis findings may be considered compatible with repetitive mechanical fatigue. The small sample size did not allow to draw robust conclusions. Future studies are needed on larger samples trying to identify the possible relationship between bruxism and anterior restorations fracture.

## 1 Introduction

A harmonious smile cannot be achieved if additional conditions, such as microdontia, peg-shaped teeth, transposition of canines instead of premolars, and agenesis, are present (1). Such alterations appear to be caused by the interaction of genetic, epigenetic and environmental factors during dental development (2). In addition, the presence of diastemas, defined as interdental spaces >0.5 mm and normally present in children, is considered a cause of blemishes if present in adults following the eruption of permanent canines (2). Therefore, these clinical situations often require, after orthodontic treatment, finalization by means of direct additive restorations. These solutions represent an aesthetic, functional and biologically effective treatment option for shape modification and diastema closure with clinically promising survival rates (3). Moreover, it is undoubtedly the most economical, most conservative, and a reversible therapeutic modality (4).

Direct restorations have been largely employed to restore anterior teeth due to their low cost and less need for the removal of sound tooth substance when compared to indirect restorations, as well as to their acceptable clinical performance. However, it is crucial to consider the patient as a whole: although additive restorations provide a therapeutic solution with strong aesthetic value, the patient's function, flawed habits, and parafunctions cannot be ignored (2).

Bruxism is currently defined as “repetitive jaw-muscle activity characterized by clenching and grinding of the teeth and/or by bracing and thrusting of the mandible” and specified as either sleep bruxism (SB) or awake bruxism (AB) (5, 6). SB is currently described as masticatory muscle activity during sleep, characterized as rhythmic (phasic) or nonrhythmic (tonic), and distinct from movement or sleep disorders in otherwise healthy individuals (6).

SB usually involves both teeth clenching and grinding, with no significant gender difference. Its prevalence is estimated at 21%, with a decrease in frequency as age increases (7). Its diagnosis is currently performed according to the Standardized Tool for the Assessment of Bruxism (STAB), which is multidimensional diagnostic tool for the evaluation of bruxism through a patient-reported, clinical, and instrumental approach (Axis A) combined with an evaluation of potential aetiology/comorbidities (Axis B) (5, 6). Thus, the diagnosis is based on a comprehensive investigation on self-awareness, masticatory muscle fatigue and pain, tooth wear, fractured restorations, or linea alba on cheeks, and on an instrumentally based assessment of the masticatory muscle events and work, through instrumental approaches such as polysomnography, or electromyographic and electrocardiographic data (6, 8, 9).

Additive direct restorations in anterior teeth have more failures than those in posterior teeth, and the reported annual failure rate of composite resin restorations ranges from 0 to 4.1%, with fracture of the restoration being the main cause of failure (10). SB seems to be associated to occlusal loading that may exceed physio-structural limits leading to negative mechanical consequences on masticatory muscles, temporomandibular joint, and occlusion (11). The bite force commonly ranges from 50 to 300N and it can increase up to 1200N in patients who have clenching or grinding teeth (12).

Some studies have tried to identify the bruxism as a risk factor for crack or fracture of natural teeth or restorations (13, 14). Laske et al. (15) investigated the possible risk factors for failure across 31,472 restorations and found that the presence of SB resulted in a higher risk for restoration fracture/wear. On the other hand, the intuitive hypothesis that bruxism would increase the risk of restoration for severe tooth wear was not confirmed by previous studies (12, 16). Thus, no clear evidence on the relationship between bruxism and restoration failure emerged from the scientific literature. Probably, the main limitation was related to bruxism diagnosis and severity assessment. Indeed, until the new consensus came out, bruxism was mainly seen as the act of clenching and grinding of the teeth and diagnosis was mainly based on the presence of presence of tooth wear.

Thus, the present study aimed at evaluating the influence of bruxism on post orthodontic direct anterior restorations integrity.

## 2 Materials and methods

### 2.1 Study design and study setting

In this retrospective study, patients were recruited at the School of Dentistry of the University of Turin, Turin, Italy. The recall of the patients started in January 2023 and ended in December 2025, after 5-year follow-up from the intervention.

The study protocol was approved by the Institutional Ethics Committee (Città della Salute e della Scienza di Torino, approval no. DS_00071_2018). All participants were asked to carefully read and sign an informed consent and were free to withdraw from the study at any time. The described protocol was conducted in accordance with the recommendations of the revised Declaration of Helsinki for investigations with human subjects. Moreover, the study was performed in accordance with the STrengthening the Reporting of OBservational studies in Epidemiology (STROBE) Guidelines.

### 2.2 Participants

We recruited adult subjects referred to the Department of Cariology and Operative Dentistry of the University of Turin, Turin, Italy, who received additive composite restorations in maxillary anterior teeth. The restorations were performed by an experienced clinician (NS) after an orthodontic treatment conducted at the Department of Orthodontics of the same School of Dentistry.

We considered for inclusion only subjects who reported “failure” (when the restoration was fractured, making repair impossible).

Controls were recruited among the patients who reported success of the additive composite restorations. The same inclusion and exclusion criteria were applied to the control group.

Inclusion criteria were as follow: adult subjects; previous orthodontic treatment in retention with upper and lower Hawley appliances (no interferences on the subject's occlusion); slight discrepancy of symmetry between upper and lower midline after orthodontic treatment; single or multiple diastema among maxillary anterior teeth; Bolton discrepancy (inter-arch tooth size discrepancies); malformed teeth (such as peg-shaped lateral incisors) needing reshaping; no active periodontal or pulpal disease; good oral hygiene (Full Mouth Plaque Score - FMPS < 20%).

Exclusion criteria were as follow: systemic disease; history or current treatment of neurological disorders (e.g., epilepsy), or psychiatric disorders; inadequate oral hygiene; sleep disorders (e.g., sleep apnea); using medications or substances known to influence bruxism were excluded (e.g., selective serotonin reuptake inhibitors, noradrenaline-selective reuptake inhibitors, cocaine, caffeine, nicotine, alcohol, clonazepam, diazepam, gabapentin); decays during orthodontic treatment, gingival/periodontal disease; uncontrolled parafunction; previous endodontic or restorative treatments; absence of antagonist teeth.

### 2.3 Intervention

#### 2.3.1 Restorative procedure at the end of orthodontic treatment

Within 30 days from the end of the orthodontic treatment, all patients underwent a pre-operative phase. It consisted of professional oral hygiene, instruction and motivation to a proper home oral hygiene, and a radiographic evaluation. Maxillary and mandibular impressions were taken in order to obtain diagnostic models. Then, a wax-up was made to design a silicone guide for clinical procedures and, finally, to make a mock-up.

A nanohybrid composite (ClearFil Majesty ES-2, Kuraray, Tokyo, Japan) and a three-step adhesive system, etch and rinse (Optibond FL, Kerr, USA) were used by the same clinician (NS) to perform all the restorations. Regarding diastema closure and tooth reshaping, the restorations were performed as symmetrically as possible. All restorations were performed according to according to Comba et al. (17).

Treatment was completed by informing patients about oral hygiene measures for cleaning the new restorations with a toothbrush and floss, and intraoral photographs were taken to facilitate follow-up evaluations.

#### 2.3.2 Clinical evaluation at follow-up

At follow-up, two calibrated examiners evaluated the restorations according to the modified United States Public Health Service (USPHS) criteria, as described by Comba et al. (17).

The dentists were trained and calibrated before the evaluation began. Cohen's kappa statistic was used to calculate inter-observer agreement. Excellent intraobserver (kappa values of 0.76 and 0.79) and inter-observer (kappa value of 0.81) agreement was found in this study. Patients who reported failure (when the restoration was lost, making repair impossible) were included in the study group.

Thus, restorations with less damaging events (such as minor composite fractures, marginal gaps, chipping, colour/surface deterioration, and caries) occurred or restorations having no failure or unfavourable event were excluded from the study. In addition, restorations from patients who reported problems not related to the restorative treatment, such as diastema relapse, were also excluded.

Controls were recruited among the patients who reported success of the additive composite restorations, matched for age and sex.

#### 2.3.3 Scanning electron microscopy (SEM) evaluation at follow-up

The precise origin of the failure (crack initiation) was identified using a specialized failure analysis protocol as described by Scherrer et al. (18). Specifically, the identification of the crack's origin and its direction of propagation is possible analysing the characteristic fractographic markers (hackle lines, arrest lines and wake hackle). This fractographic evidence can allow clinician to understand the causes of the failure and thus helping in preventing future failures.

Patients in the study group underwent silicone impressions of the restored teeth, and replicas were made using an epoxy resin. A one-stage impression was taken using a double viscosity polyvinyl-siloxane (one-step heavy/light-body silicone impression) in a perforated metal stock tray; specifically air-drying, cleaning with alcohol 90%, air-drying, extra-light body silicone (Imprint II Garant Light Body, 3M ESPE, USA) on vestibular and interproximal surfaces, heavy silicon impression (Express™ 2 Penta™ H, 3M ESPE, USA) were performed.

Subsequently, the impressions were sent to the University Clinics of Dental Medicine at the University of Geneva, where they were cast to obtain epoxy resin models (Sherapolan 2:1, Shera Werkstoff-Technologie GmbH). A failure analysis was performed on these models to confirm the clinical evaluation. The molds of the selected teeth, incisors and canines, were visualized under a scanning electron microscope (SEM) (Digital SEM XL20, Philips, Amsterdam, The Netherlands) for more detailed analysis of the fractured surfaces.

To clean the templates from impurities, replicas were immersed in an ultrasonic 10% NaOCl bath for 3 min, rinsed with water, dried and then fixed on the support for the microscope. The replicas were gold-coated prior to analysis with the SEM. Magnifications up to ×500 were used to obtain higher definition images of identified crack features in selected areas of interest. Different magnification (×13, ×15, ×16, ×100, ×500) images were obtained with the following settings: WD = 26–44 mm; aperture size = 10 μm–1 mm; EHT = 100 kV; signal A = SE2.

#### 2.3.4 Bruxism evaluation at follow-up

At follow-up, a bruxism evaluation was performed according to the STAB, a multidimensional diagnostic tool for the evaluation of bruxism (5). The clinically based bruxism assessment was performed by evaluating tooth wear facets, presence of linea alba, and masseter hypertrophy. The subjects-based assessment was performed using the Oral Behavior Checklist (OBC), to assess the frequency of sleeping-state and waking-state oral behaviors and the patient self-reports of clenching or grinding during sleep or wakefulness (items A1.1 and A2.1).

Patients were classified as bruxists when at least one clinical sign was found and patient self-reports confirmed the diagnosis.

The portable device Bruxoff (Bruxoff®, Spes Medica, Battipaglia, Italy) with three channels was used for an instrumentally based assessment to acquire sEMG bilaterally from the masseter and the heart frequency (19–23).

**Figure 1:**
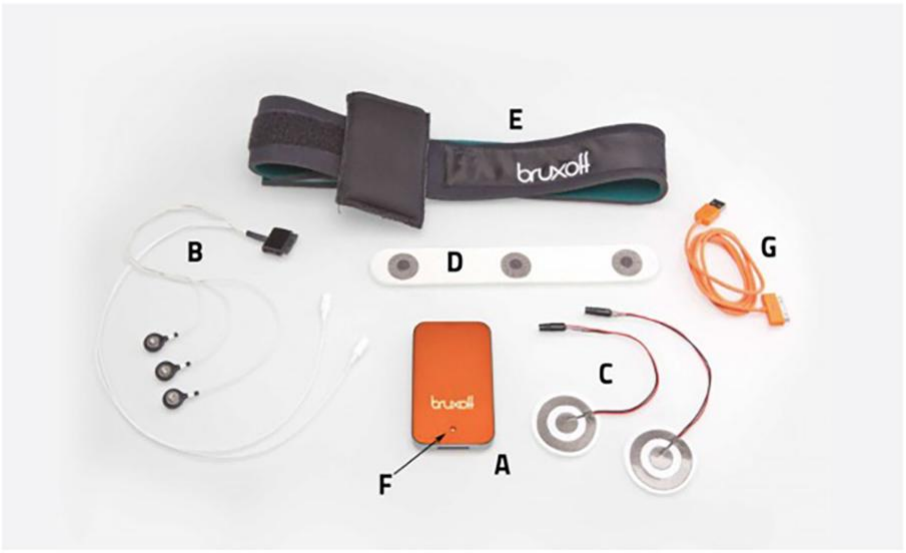
Bruxoff*®* and its components. **(A)** the electrodes connection cable, **(B)** the pair of electromyographical electrodes, **(C)** the cardiac electrodes, **(D)** the chest strap, **(E)** the start/stop button, **(F)** the USB cable, **(G)** USB, universal serial bus.

All patients received and were instructed to use the Bruxoff device (Bruxoff®, Spes Medica, Battipaglia, Italy) (19, 20). They were provided with a kit containing all necessary components for the test, including the user's manual (Figure 1). In addition, the investigators provided instructions on using the device through a demonstration to each patient. All subjects underwent two consecutive recording nights (at least 4 h of sleep per night) and only data recorded during the second night were considered for statistical analyses, as previously reported by Castroflorio et al. (8).

The device with three channels uses concentric electrodes to detect surface electromyographic signals (sEMG) from two masseter muscles and a band that incorporates three surface electrodes to detect heart rate. The EMGs from the masseter muscles was with disposable bipolar concentric electrodes (Code®, Spes Medica, Battipaglia, Italy), with a radius of 16 mm and with detection site made of AgCl.

Data from each recording were subsequently downloaded to the appropriate Bruxmeter software (Bruxmeter®, OT Biolettronica, Turin, Italy). Before starting, the subjects performed three maximum voluntary clenching (MVC) lasting 3 s each and separated by 10 s of rest. The greatest of the MVC measures was used to set the device. Specifically, the software recognizes a sleep bruxism episode a sEMG burst greater than 10% MVC preceded 1 s before by a heart rate increase of 20% with respect to the baseline (24).

The software provides a number of parameters (recording duration, bruxism index, number of bruxism episodes, the number of episodes per hour, and the total number of masseter muscle contractions, categorized as phasic, tonic, or mixed contractions, the average heart rate). Specifically, the following electromyographic variables were assessed: surface masticatory muscle activity (sMMA), bruxism index (BI), phasic contractions (PC), phasic bruxism-related contractions (PCB), tonic contractions (TC), tonic bruxism-related contractions (TCB), mixed contractions (MC), mixed bruxism-related contractions (MCB), heart rate (HR) and total masseter contraction (TMC).

The portable device has high sensitivity (92.3%) and specificity (91.6%) for sleep bruxism diagnosis when the diagnostic cut-off was set at 4 sleep bruxism episodes per hour (9). Thus, for determining the prevalence of sleep bruxism, participants diagnosed with more than 4 episodes per hour were classified as “positive”, whereas those with a “zero” score were considered “negative”.

Lastly, patients underwent a Bruxchecker assessment (21–23). The Bruxchecker® (BC) was developed by Onodera et al. (21) and is a red color-coated 0.1 mm thick polyvinyl sheet that can be fitted into the mouth with minimal discomfort. To produce them, an alginate impression (Zhermack Dental, Dentsplay Sirona, Italy) in a perforated metal tray was taken. After casting the impressions within 30 min, the plaster models were sent to the technician who made a maxillary Brux-checker template thermos-printed, according to manufacturer's instructions (Scheu-Dental GmbH, Iserlohn, Germany).

**Figure 2:**
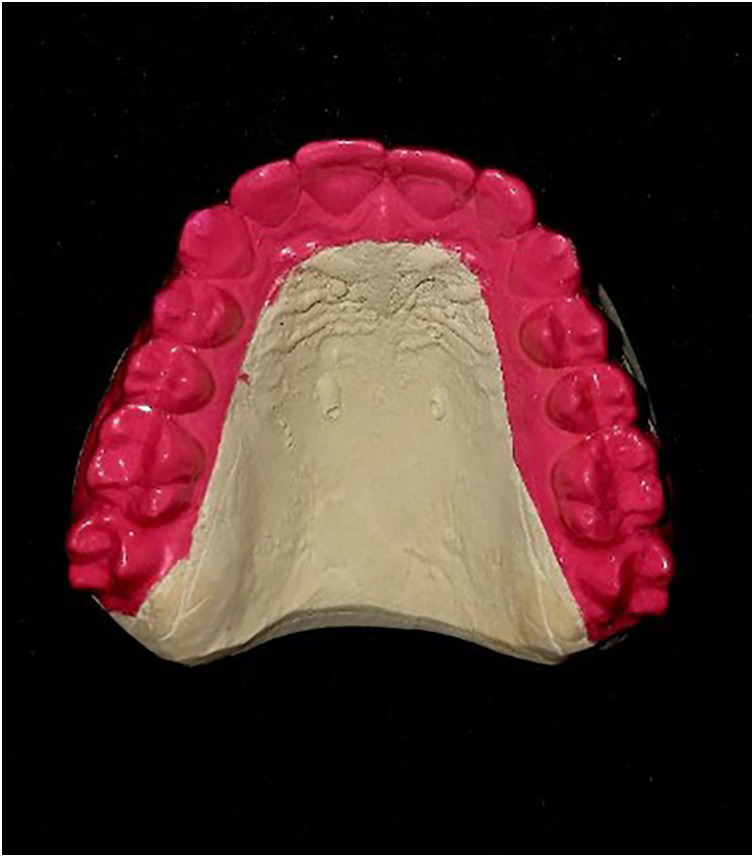
Bruxchecker*®* before wearing.

The Bruxchecker foils were 0.1 mm thick, with a circumference of 125 mm, and was composed of a polyvinyl chloride foil coated with disodium 2-benzoate (see Figure 2). A layer of biocompatible colour was applied to one side of these foils, which will be ground on the contact points between the upper and lower jaws. In the present study, patients were asked to wear the template overnight for a single night.

**Figure 3:**
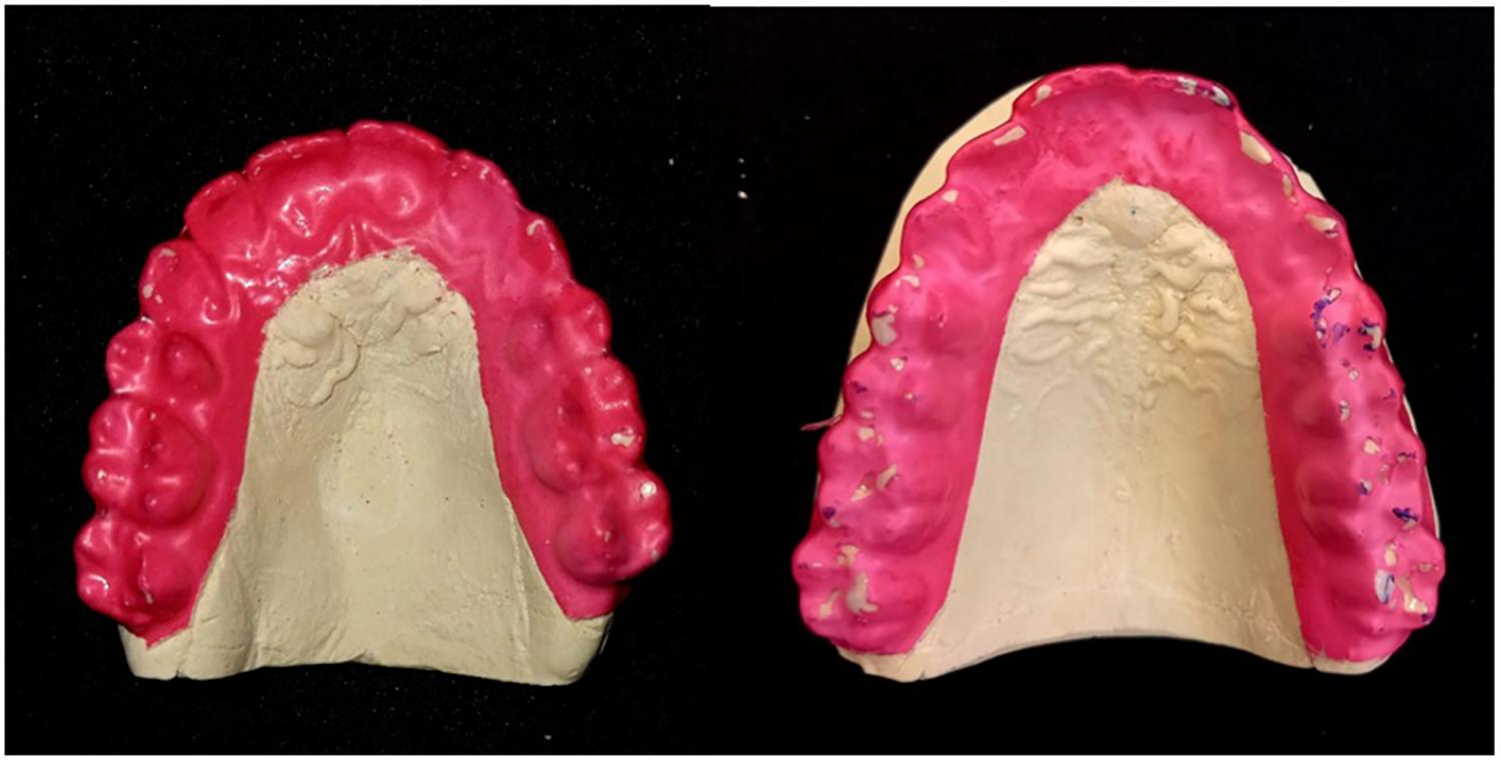
Left: example of non-bruxist patient, with light contacts on canine and molars without involvement of incisors. Right: example of bruxist patient, with contacts eccentric to intercuspal position (ICP) points and involving anterior elements.

Upon delivery, the Bruxcheckers were evaluated by a blinded operator, based on the tooth contacts corresponding to the discolored marks on the template. By purely descriptive analysis, it was determined whether the discolorations followed an eccentric or centric movement pattern with respect to the normal closing position between the two maxillary arches. It was also considered whether these contacts were occurring only at the level of the diatoric teeth or also at the level of the frontal sector, going to affect the additive restorations, affecting their integrity (see Figure 3).

### 2.4 Statistical analysis

Statistical analysis was conducted using the R-4.4.1 software (R Foundation, Vienna, Austria). The continuous variables are presented as means ± standard deviations. The accuracy of the Bruxoff device in determining the bruxism diagnosis was defined based on the level of agreement between the subjects-based and clinically based bruxism assessment according to the STAB guidelines and the BI > 4, assessed through Cohen's K. The range of variation of the K statistic is between 0 for no agreement and 1 for perfect agreement with five intermediate levels: “slight agreement” (0.01–0.20), “fair agreement” (0.21–0.40), “moderate agreement” (0.41–0.60), “substantial agreement” (0.61–0.80) and “almost perfect agreement” (0.81–0.99). Regarding the comparison of means of study and control groups as significantly different, we have performed one-way analysis of variance (or one-way ANOVA). Statistical significance was set at *p* *<* 0.05.

## 3 Results

A total 20 patients (8 males and 12 females, mean aged 25.67 ± 4.77 years) were included in the present study, 10 subjects in the study group (3 males and 7 females, mean aged 28.33 ± 5.75 years) and 10 subjects in the control group (3 males and 7 females, mean age 23.00 ± 2.10 years old).

Out of 40 restorations, 24 restorations (60%) were in female patients and 16 (40%) were in males. All the restorations were in the anterior region of the upper maxilla: 19 central incisors, 17 lateral incisors, 4 canines. The follow-up time varied from 5 to 9 years, with a mean observation period of 7.1 ± 1.2 years.

Specifically, 19 restorations were evaluated in the study group (11 were performed to correct diastema and 8 to recontour tooth shape, mean follow-up was 7.0 ± 1.6 years). Twenty-one restorations were evaluated in the control group (10 were performed to correct diastema and 11 to recontour tooth shape, mean follow-up was 7.1 ± 1.1 years).

The SEM evaluation was conducted to obtain a fractographic analysis to identify the precise origin of the failure (crack initiation). The epoxy resin replicas of the 40 anterior restorations (comprising 19 central incisors, 17 lateral incisors, and 4 canines) were analyzed at the University of Geneva using the protocol as described by Scherrer et al. (18). The time in service ranged between 5 and 9.25 years, with a mean follow-up of 6.78 ± 1.26 years. The long-term observation of fractures restorations was requested to better investigate the artefacts and falsifying.

**Figure 4:**
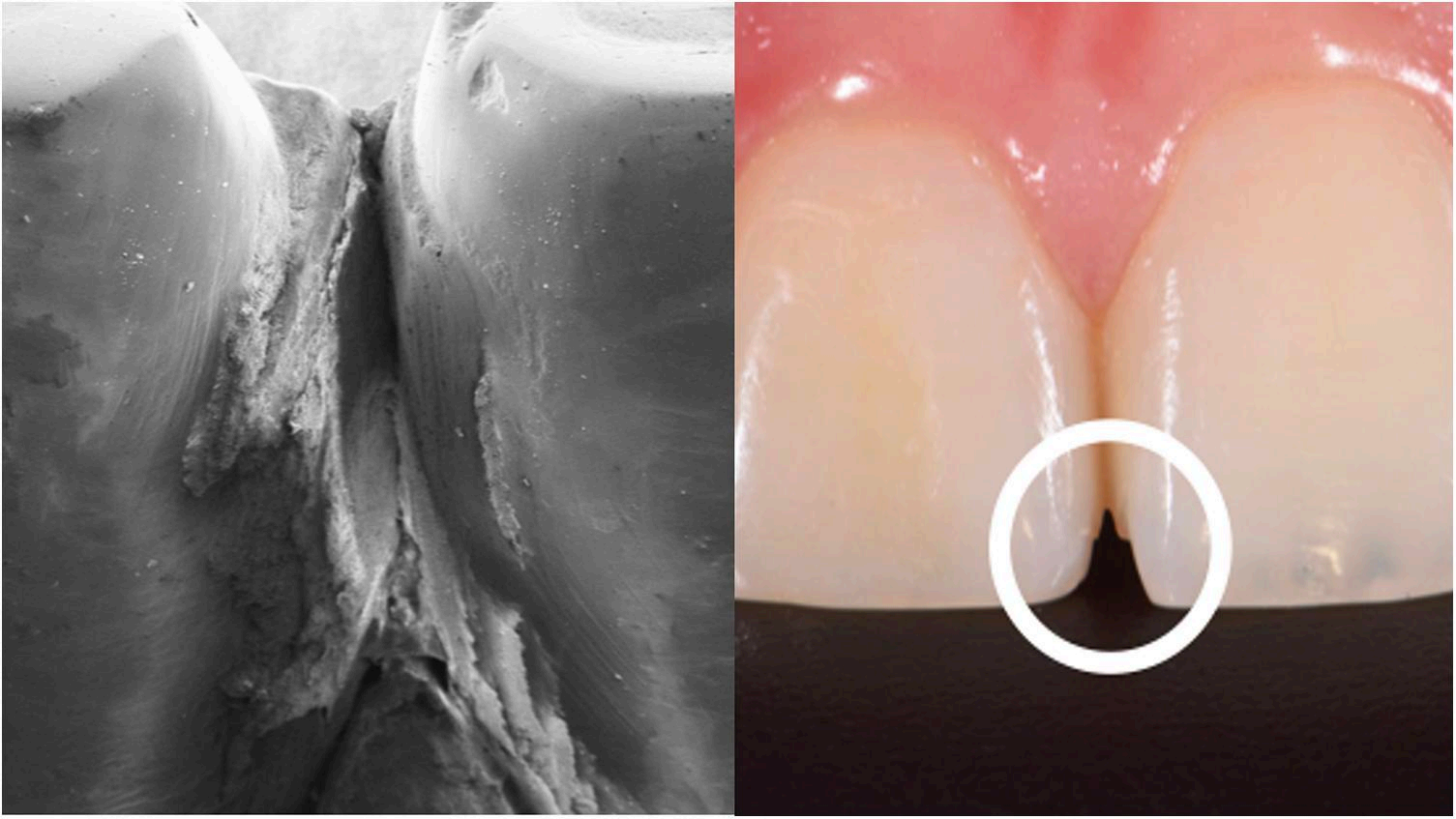
Scanning electron micrograph of rubbing surface shows surface crack running along the incisal edge.

**Figure 5:**
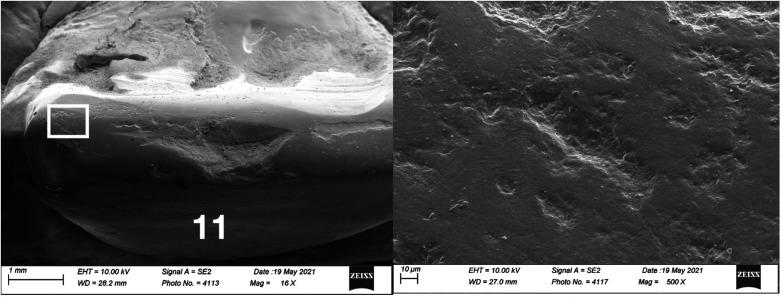
Macroscopic appearance of a Stage II fatigue fracture: smooth surface covered by beach marks.

Results revealed that the fractures originated at the site of traumatic contact with the antagonist tooth (Figures 4, 5). Thus, the findings were compatible with repetitive mechanical fatigue.

The macroscopic lines shown in Figure 4 represent the crack propagation front at the locations where there have been load amplitude changes or environmental variations.

Beach marks usually arch towards the outer edges of the fractured body. This is due to the faster crack growth rate in the central zone of thickness, which is in plane strain. The Stage II fractured surfaces have a smooth appearance, often with a faint coloration due to the interaction with the environment; they usually have ratchet steps resulting from initial crack propagating in planes at different levels, and the orientation of the fracture plane is generally perpendicular to the direction of the maximum principal stress. Figure 5 shows the typical aspect of a Stage II fatigue fracture displaying the characteristics described above. The fractured surface extension in Stage II depends on the maximum stress and the stress concentration level.

According to the subjects-based and clinically based bruxism assessment, 60% of subjects in the study group and 30% of subjects in the control group were diagnosed as bruxists. According to the instrumentally based assessment (BI values > 4), 70% of subjects in the study group and 40% of subjects in the control group were diagnosed as bruxists. The agreement between subjects-based and clinically based bruxism assessment and instrumentally based assessment (BI values > 4) was good (kappa value of 0.8125).

All the recruited participants completed the registrations with the Bruxoff device without reporting any detachment of the electrodes. In addition, all subjects reported no considerable sleep interruption that might have influenced the outcomes.

The average duration of the sleep examination was 7 h and 7 min ± 2 h and 1 min. Specifically, the average duration was 6 h and 42 min ± 1 h and 33 min in the study group, and 7 h and 31 min ± 2 h and 29 min in the control group. The electromyographic variables assessed using the Bruxoff device in study and control groups are showed in Table 1. The mean BI was 6.30 ± 5.15 in the study group and 2.65 ± 1.91 in the control group.

**Table 1:** Electromyographic variables assessed using the Bruxoff device in study and control groups.

Variable	Study group		Control group		*p*-value
	Mean	SD	Mean	SD	
sMMA	38.00	30.19	22.67	15.06	0.29
BI	6.30	5.15	2.65	1.91	0.23
PC	47.67	29.53	15.50	19.18	0.05*
PCB	6.33	4.55	7.33	7.31	0.78
TC	75.17	58.44	17.67	18.76	0.04*
TCB	12.00	15.97	7.67	5.32	0.54
MC	7.67	3.72	3.17	2.99	0.04*
MCB	1.17	1.60	1.50	1.64	0.73
HR	73.17	10.83	64.50	7.69	0.14
TMC	269.5	80.80	55.67	50.06	0.0003**

Continuous variables were reported as means ± standard deviations. sMMA, surface masticatory muscle activity; BI, bruxism index; PC, phasic contractions; PCB, phasic bruxism-related contractions; TC, tonic contractions; TCB, tonic bruxism-related contractions; MC, mixed contractions; MCB, mixed bruxism-related contractions; HR, heart rate; TMC, total masseter contraction.

*p-value < 0.05; **p-value < 0.005.

**Figure 6:**
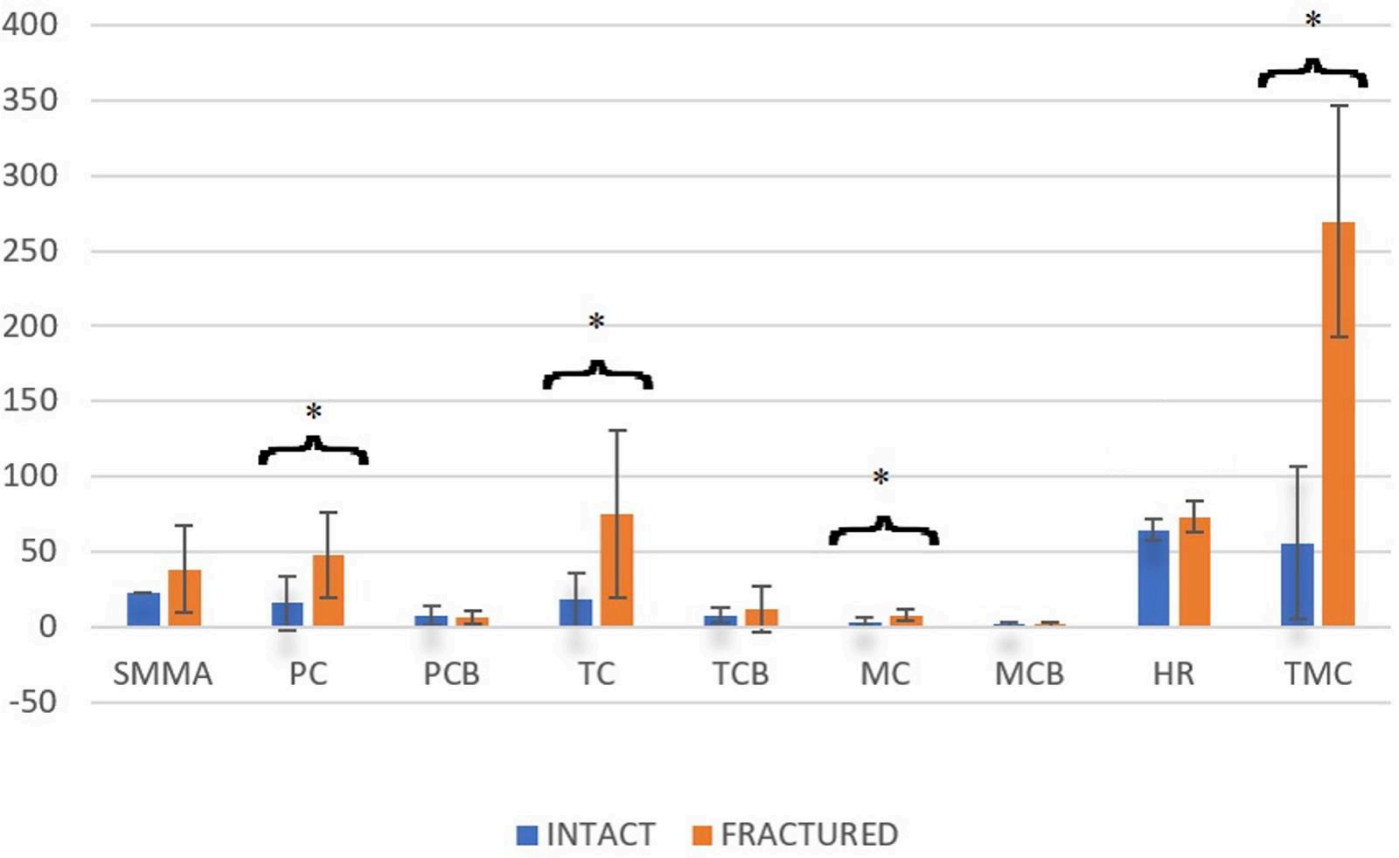
The graph represents the averages of the electromyographic variables assessed using the Bruxoff device considered between the two groups (blue = intact; orange = fractures). Variables marked with an asterisk were found to be statistically significant.

The Figure 6 showed the graphic representing the averages of the electromyographic variables assessed using the Bruxoff device, considered between the two groups (orange = study group; blue = control group). Variables marked with an asterisk were found to be statistically significant.

The electromyographic variables that resulted to be significantly different between groups: PC (*p*-value = 0.003), TC (*p*-value = 0.003), MC (*p*-value = 0.002), and TMC (*p*-value = 0.000) (Table 1). BI was not statistically significant (*p*-value = 0.23).

The Bruxchecker analysis revealed that the occlusal patterns of the patients with intact restorations (Control group) appeared to be very similar. In 5 we appreciate contacts with discoloration at the level of the upper canines: sign of canine guidance disclosing the sector of posterior teeth; in 4 others we also appreciate slight contacts with centric pattern on premolars and molars. Only one Bruxchecker template from patients with intact restorations showed contacts on the incisal margin and palatine surfaces of the central incisors.

Analyzing the Bruxcheckers of the 10 patients with fractured direct additive restorations (study group), the morphology of the contacts in all templates showed discoloration on both the incisors and diatoric teeth, sign that the patient's occlusion or nocturnal bruxism activity might have affected the incisal margins or palatine surfaces of the anterior teeth.

## 4 Discussion

During the last decade, the diffusion of sEMG has been increased among researchers and clinicians involved in in dentistry field (25). Its utility in temporomandibular disorders (TMDs) and orofacial pain has always been questioned because of the inability to add significant information in addition to the clinical assessment. On the other hand, it represents the main type of instrumentally based assessment strategy for bruxism (26). In this context, a meta-analysis by Casett et al. (26) was conducted to evaluate the diagnostic validity of clinical assessment, questionnaires, and portable diagnostic devices in comparison with polysomnography, considered as the gold standard method for SB diagnosis. The authors concluded that portable diagnostic devices showed the best validity of all evaluated methods.

Moreover, according to the Standardized Tool for the Assessment of Bruxism (STAB) (5, 6), the subjects-based and clinically based bruxism assessment should be implemented through an instrumentally based assessment that includes sleep-time EMG. Considering the high variability in terms of intensity and duration of the bruxism events occurring during sleep, several studies investigated the ideal EMG criteria for detecting SB (8, 19, 20). As suggested by the STAB, the devices should recognize as SB episode a sEMG burst greater than 10% MVC. Indeed, the MVC can be as a potentially useful reference to discriminate SB events, considering the high intraindividual reproducibility (27, 28). In addition, the Bruxoff device identifies MVC increase accompanied by a 20% increase in the heart rate with respect to the baseline (24).

Despite the few numbers of research on instrumental based assessment is available in the scientific literature, these devices can provide bias-free information about the frequency of bruxism and should represent the future. The main limitations in Bruxoff utilization are related to the high economic cost, potential need for multiple nights, and patient compliance due to the difficulty to perform properly conducted registrations at home.

In the present study, all the recruited participants completed the registrations with the Bruxoff device, without reporting any detachment of the electrodes. In addition, all subjects reported no considerable sleep interruption that might have influenced the outcomes.

The analysis of bruxism by Bruxoff pointed out that there are statistically significant differences between the group of patients with fractured restorations and the group with intact restorations in the variables of: phasic contractions (PC), tonic contractions (TC), mixed contractions (MC), heart rate (HR) and total masseter contractions (TMC). The group of patients with fractured restorations had significantly higher masseter muscle activity than the group of patients with intact restorations. However, although BI was higher in study group compared to controls (6.30 vs. 2.65), the difference was not statistically significant (*p*-value = 0.23).

It should be considered the high risk of Type I error due to the multiplicity of electromyographic variables analyzed (PC, TC, MC, HR, TMC). Thus, results should be evaluated with caution.

Several factors can influence restorations success, including oral hygiene habits, risk of caries, occlusal relationships, parafunctional habits, location of the restoration, size and physical properties of the used material and the most common causes of failure of direct composite restorations are secondary caries, wear, and fractures (15).

Bruxism may produce repetitive high-magnitude loading and increased sliding contacts, accelerating abrasional wear and promoting fatigue-related surface degradation (29). As stated in many studies, bruxism may play a significant role in the failure of direct restorations (30).

The chewing forces may be higher in subjects with bruxism as the loads can be up to six times greater than normal forces. Moreover, while chewing forces are primarily vertical, they can also be horizontal during SB movements (31).

According to Demarco et al. (14), clenching and grinding activities may cause fatigue in the tooth-restoration complex, resulting in fracture over time. The tooth-composite interface represents the weakest portion of composite restorations (32, 33). To date, no adhesive system seems to be able to eliminate marginal losses in the long term (32). Consequently, it is easy to understand how bruxism loads that are transmitted on elements with additive restorations can compromise the integrity of the composite-tooth interface. In addition, as is stated by Moura et al. (34), the involvement of incisal angles in Class IV restorations causes stresses that are not evident in other cavity configurations, putting a strain on the composite-tooth interface. Class IV restorations are subject to high loads from the antagonist tooth, with extra-axial direction, and bruxism stress can compromise their longevity.

Studies on the fatigue of resin-based composite materials demonstrated that parafunctional cyclic loading, including repetitive occlusal overload or bruxism, could induce progressive crack initiation and propagation within the polymer matrix and at the filler–matrix interface (35, 36). Fractographic analyses performed in these studies identified characteristic features of Stage II fatigue fracture, including stable crack growth regions, fatigue striations, arrest lines, and hackle markings oriented perpendicular to the direction of tensile stress (37). Drummond (35) reported that cyclic loading promotes the accumulation of subsurface microstructural damage, eventually resulting in catastrophic fracture after repeated stress cycles. Similarly, Drummond (36) described fatigue degradation as a time-dependent process involving interfacial debonding, matrix softening, and filler particle dislodgement under repeated loading conditions. These observations were later corroborated by Kruzic et al. (37), who demonstrated that the fatigue crack propagation behavior of dental composites closely resembles that of other brittle particulate-filled materials subjected to cyclic mechanical stresses. The presence of Stage II fatigue fracture morphology therefore supports the hypothesis that repetitive parafunctional loading plays a critical role in the long-term structural degradation and failure of composite resin restorations (38, 39).

However, direct additive restorations are the best and most conservative treatment choice in young patients. The material composition can influence the long-term aesthetical performance of resin composites. In the present study, to overcome aesthetic problems related to microhybrid composites, nanohybrid resins were used. Thanks to the hybrid structure and to their excellent ability to be polished, the resins allowed clinician to obtain higher wear resistance and mechanical strength with improved aesthetics (40, 41). An *in vivo* study showed that nano and a nanohybrid composite direct composite restorations showed a good clinical durability, reporting a high survival rate after 4 years of clinical function (42). Similarly, Comba et al. (17) evaluated direct additive composite restorations performed to correct anterior teeth discrepancies after orthodontic treatment at long follow-up (up to 10 years). The authors analyzed a total of 169 restoration and showed an overall survival rate of additive restoration of 2.59%, with chipping of the material and composite wear as the most frequent adverse event. Although restorations are destined to fail in a shorter or longer time, they accompany the young patient toward adulthood when the option of indirect restorations can, then, be considered.

The Bruxcheckers analyses revealed discoloration on both the incisors and diatoric teeth, sign that the patient's occlusion or nocturnal bruxism activity might have affected the incisal margins or the palatine surfaces of the anterior teeth. However, it should be considered that the presence of contacts in the anterior region (incisors) could be also related to a loss of the canine guidance due to wear in bruxist patients.

The main limitation of the present study was the small sample size that could not allow for the control of confounding variables or the drawing of robust conclusions. The high risk of Type I error due to the multiplicity of electromyographic variables analyzed (PC, TC, MC, HR, TMC) should be considered. Manual verification of the EMG traces was not performed.

The follow-up time was different among participants, and this could have affected the restorations load. The bruxism assessment at follow-up can only allow identification of an association between current electromyographic findings and previous restoration fracture.

All restorations were performed by a single clinician who used the same nanohybrid composite and adhesive system. This allowed to reduce the internal technical variability, but, on the other hand, it limited the generalizability of the results to the global dental community. As we included only post-orthodontic patients, occlusal characterization of the sample was not reported.

Lastly, we did not assess the potential aetiology/comorbidities according to the STAB Axis B.

On the other hand, this is the first study evaluating the influence of bruxism on post orthodontic direct anterior restorations integrity in the scientific literature. Future studies are needed on larger samples trying to identify the possible relationship between bruxism and anterior restorations failure, also evaluating the differences in outcome in relation to the various types of bruxism activities.

## 5 Conclusions

At follow-up, patients with fractured anterior restorations showed significantly higher masseter muscle activity, evaluated in terms of phasic contractions, tonic contractions, mixed contractions, and total masseter contractions using a portable sEMG portable device. The fractographic analysis revealed that the fractures originated in a site of traumatic contact with the antagonist tooth. Thus, the findings may be considered compatible with repetitive mechanical fatigue.

The small sample size did not allow to draw robust conclusions and future studies are needed on larger samples trying to identify the possible relationship between bruxism and anterior restorations failure.

## Data Availability

The raw data supporting the conclusions of this article will be made available by the authors, without undue reservation.

## References

[1] Peck S Peck L Kataja M. Prevalence of tooth agenesis and peg-shaped maxillary lateral incisor associated with palatally displaced canine (PDC) anomaly. Am J Orthod Dentofacial Orthop. (1996) 110(4):441–3. 10.1016/s0889-5406(96)70048-3 8876497

[2] Khalaf K Miskelly J Voge E Macfarlane TV. Prevalence of hypodontia and associated factors: a systematic review and meta-analysis. J Orthod. (2014) 41(4):299–316. 10.1179/1465313314Y.0000000116 25404667

[3] Wolff D Kraus T Schach C Pritsch M Mente J Staehle HJ. Recontouring teeth and closing diastemas with direct composite buildups: a clinical evaluation of survival and quality parameters. J Dent. (2010) 38(12):1001–9. 10.1016/j.jdent.2010.08.017 20826192

[4] Korkut B Türkmen C. Longevity of direct diastema closure and recontouring restorations with resin composites in maxillary anterior teeth: a 4-year clinical evaluation. J Esthet Restor Dent. (2021) 33(4):590–604. 10.1111/jerd.12697 33354867

[5] Manfredini D Ahlberg J Aarab G Bender S Bracci A Cistulli PA. Standardised tool for the assessment of bruxism. J Oral Rehabil. (2024) 51(1):29–58. 10.1111/joor.13411 36597658

[6] Manfredini D Ahlberg J Lavigne GJ Svensson P Lobbezoo F. Five years after the 2018 consensus definitions of sleep and awake bruxism: an explanatory note. J Oral Rehabil. (2023) 51(3):623–4. 10.1111/joor.13626 37994212

[7] Zieliński G Pająk A Wójcicki M. Global prevalence of sleep bruxism and awake bruxism in pediatric and adult populations: a systematic review and meta-analysis. J Clin Med. (2024) 13(14):4259. 10.3390/jcm13144259 39064299 PMC11278015

[8] Castroflorio T Bargellini A Rossini G Cugliari G Deregibus A Manfredini D. Agreement between clinical and portable EMG/ECG diagnosis of sleep bruxism. J Oral Rehabil. (2015) 42(10):759–64. 10.1111/joor.12320 26059761

[9] Castroflorio T Deregibus A Bargellini A Debernardi C Manfredini D. Detection of sleep bruxism: comparison between an electromyographic and electrocardiographic portable holter and polysomnography. J Oral Rehabil. (2014) 41(3):163–9. 10.1111/joor.12131 24417585

[10] Smales RJ Berekally TL. Long-term survival of direct and indirect restorations placed for the treatment of advanced tooth wear. Eur J Prosthodont Restor Dent. (2007) 15(1):2–6. PMID: . 17378451

[11] Manfredini D Winocur E Guarda-Nardini L Lobbezoo F. Self-reported bruxism and temporomandibular disorders: findings from two specialised centres. J Oral Rehabil. (2012) 39(5):319–25. 10.1111/j.1365-2842.2011.02281.x 22251149

[12] Hamburger JT Opdam NJ Bronkhorst EM Huysmans MC. Indirect restorations for severe tooth wear: fracture risk and layer thickness. J Dent. (2014) 42(4):413–8. 10.1016/j.jdent.2013.10.003 24120523

[13] Lempel E Lovász BV Meszarics R Jeges S Tóth Á Szalma J. Direct resin composite restorations for fractured maxillary teeth and diastema closure: a 7 years retrospective evaluation of survival and influencing factors. Dent Mater. (2017) 33(4):467–76. 10.1016/j.dental.2017.02.001 28256273

[14] Demarco FF Corrêa MB Cenci MS Moraes RR Opdam NJ. Longevity of posterior composite restorations: not only a matter of materials. Dent Mater. (2012) 28(1):87–101. 10.1016/j.dental.2011.09.003 22192253

[15] Laske M Opdam NJM Bronkhorst EM Braspenning JCC Huysmans MCDNJM. Risk factors for dental restoration survival: a practice-based study. J Dent Res. (2019) 98(4):414–22. 10.1177/0022034519827566 30786222

[16] Hamburger JT Opdam NJ Bronkhorst EM Kreulen CM Roeters JJ Huysmans MC. Clinical performance of direct composite restorations for treatment of severe tooth wear. J Adhes Dent. (2011) 13(6):585–93. 10.3290/j.jad.a22094 21935514

[17] Comba A Vergano EA Baldi A Alovisi M Pasqualini D Castroflorio T. 5-year retrospective evaluation of direct composite restorations in orthodontically treated patients. J Dent. (2021) 104:103510. 10.1016/j.jdent.2020.103510 33130052

[18] Scherrer SS Quinn JB Quinn GD Wiskott HW. Fractographic ceramic failure analysis using the replica technique. Dent Mater. (2007) 23(11):1397–404. 10.1016/j.dental.2006.12.002 17270267 PMC2077838

[19] Yanez-Regonesi F Eisa E Judge S Carlson C Okeson J Moreno-Hay I. Diagnostic accuracy of a portable device (Bruxoff®) to measure sleep bruxism. J Oral Rehabil. (2023) 50(4):258–66. 10.1111/joor.13416 36648354

[20] Saracutu OI Pollis M Bulferetti LB Kaya E Cagidiaco FE Ferrari M. Comparison between two different registration protocols for the count of sleep bruxism events in a sample of healthy individuals. Clin Oral Investig. (2025) 29(7):365. 10.1007/s00784-025-06394-2 40593237 PMC12213913

[21] Onodera K Kawagoe T Sasaguri K Protacio-Quismundo C Sato S. The use of a bruxchecker in the evaluation of different grinding patterns during sleep bruxism. Cranio. (2006) 24(4):292–9. 10.1179/crn.2006.045 17086859

[22] Ustrell-Barral M Zamora-Olave C Khoury-Ribas L Rovira-Lastra B Martinez-Gomis J. The BruxChecker system for quantitatively assessing sleep bruxism at the dental level: reliability, reference values and methodological considerations. J Oral Rehabil. (2025) 52(7):979–90. 10.1111/joor.13959 40247454 PMC12162415

[23] Yakkaphan P Tangpothitham S Srisomphot W Khaoropphan S Nitithamakul W Lertniti P. Accuracy of the BruxChecker oral device for the assessment of sleep bruxism. Br Dent J. (2026). 10.1038/s41415-025-9203-4 41545654

[24] Deregibus A Castroflorio T Bargellini A Debernardi C. Reliability of a portable device for the detection of sleep bruxism. Clin Oral Investig. (2014) 18(8):2037–43. 10.1007/s00784-013-1168-z 24374575

[25] Zieliński G Gawda P. Surface electromyography in dentistry-past, present and future. J Clin Med. (2024) 13(5):1328. 10.3390/jcm13051328 38592144 PMC10931581

[26] Casett E Réus JC Stuginski-Barbosa J Porporatti AL Carra MC Peres MA. Validity of different tools to assess sleep bruxism: a meta-analysis. J Oral Rehabil. (2017) 44(9):722–34. 10.1111/joor.12520 28477392

[27] Jung JK Im YG. Can the subject reliably reproduce maximum voluntary contraction of temporalis and masseter muscles in surface EMG? Cranio. (2025) 43(3):380–9. 10.1080/08869634.2022.2142234 36334278

[28] Im YG Han SH Park JI Lim HS Kim BG Kim JH. Repeatability of measurements of surface electromyographic variables during maximum voluntary contraction of temporalis and masseter muscles in normal adults. J Oral Sci. (2017) 59(2):233–45. 10.2334/josnusd.16-0434 28637983

[29] Manfredini D Poggio CE. Prosthodontic planning in patients with temporomandibular disorders and/or bruxism: a systematic review. J Prosthet Dent. (2017) 117(5):606–13. 10.1016/j.prosdent.2016.09.012 27836142

[30] van de Sande FH Collares K Correa MB Cenci MS Demarco FF Opdam N. Restoration survival: revisiting patients' risk factors through a systematic literature review. Oper Dent. (2016) 41(S7):S7–26. 10.2341/15-120-LIT 27689931

[31] Lempel E Lovász BV Bihari E Krajczár K Jeges S Tóth Á. Long-term clinical evaluation of direct resin composite restorations in vital vs. endodontically treated posterior teeth—retrospective study up to 13 years. Dent Mater. (2019) 35(9):1308–18. 10.1016/j.dental.2019.06.002 31278018

[32] De Munck J Van Landuyt K Peumans M Poitevin A Lambrechts P Braem M. A critical review of the durability of adhesion to tooth tissue: methods and results. J Dent Res. (2005) 84(2):118–32. 10.1177/154405910508400204 15668328

[33] Baldi A Rossi T Comba A Vergano EA Montrella R Pampaloni B. The ability of highly-filled flowable composites in preventing marginal gap in class V restorations: an optical coherence tomography study. BMC Oral Health. (2025) 25(1):619. 10.1186/s12903-025-05970-y 40269874 PMC12020335

[34] Moura FR Romano AR Lund RG Piva E Rodrigues JSA Demarco FF. Three-year clinical performance of composite restorations placed by undergraduate dental students. Braz Dent J. (2011) 22(2):111–6. 10.1590/s0103-64402011000200004 21537583

[35] Drummond JL. Cyclic fatigue of composite restorative materials. J Oral Rehabil. (1989) 16(6):509–20. 10.1111/j.1365-2842.1989.tb01372.x 2809853

[36] Drummond JL. Degradation, fatigue, and failure of resin dental composite materials. J Dent Res. (2008) 87(8):710–9. 10.1177/154405910808700802 18650540 PMC2561305

[37] Suresh S. Fatigue of Materials. 2nd ed. Cambridge: Cambridge University Press (1998).

[38] Kruzic JJ Arsecularatne JA Tanaka CB Hoffman MJ Cesar PF. Recent advances in understanding the fatigue and wear behavior of dental composites and ceramics. J Mech Behav Biomed Mater. (2018) 88:504–33. 10.1016/j.jmbbm.2018.08.008 30223214

[39] Schijve J. Fatigue of Structures and Materials. Dordrecht: Springer (2009). 10.1007/978-1-4020-6808-9

[40] Comba A Baldi A Carossa M Paolone G Stura I Migliaretti G. A three-step etch-and-rinse vs a universal adhesive in nanohybrid composite anterior restorations: a retrospective clinical evaluation. J Adhes Dent. (2023) 25:87–97. 10.3290/j.jad.b4043039 37093568 PMC11734306

[41] Endo T Finger WJ Kanehira M Utterodt A Komatsu M. Surface texture and roughness of polished nanofill and nanohybrid resin composites. Dent Mater J. (2010) 29(2):213–23. 10.4012/dmj.2009-019 20379033

[42] Demirci M Tuncer S Öztaş E Tekçe N Uysal Ö. A 4-year clinical evaluation of direct composite build-ups for space closure after orthodontic treatment. Clin Oral Investig. (2015) 19(9):2187–99. 10.1007/s00784-015-1458-8 25802222

